# Comparison of simultaneous saccharification and fermentation with LPMO-supported hybrid hydrolysis and fermentation

**DOI:** 10.3389/fbioe.2024.1419723

**Published:** 2024-07-11

**Authors:** Chaojun Tang, Adnan Cavka, Mai Bui, Leif J. Jönsson

**Affiliations:** ^1^ Department of Chemistry, Umeå University, Umeå, Sweden; ^2^ Sekab, Örnsköldsvik, Sweden

**Keywords:** lignocellulose bioconversion, lytic polysaccharide monooxygenase, LPMO, cellulase, simultaneous saccharification and fermentation, hybrid hydrolysis and fermentation, yeast

## Abstract

Enzymatic saccharification is used to convert polysaccharides in lignocellulosic biomass to sugars which are then converted to ethanol or other bio-based fermentation products. The efficacy of commercial cellulase preparations can potentially increase if lytic polysaccharide monooxygenase (LPMO) is included. However, as LPMO requires both a reductant and an oxidant, such as molecular oxygen, a reevaluation of process configurations and conditions is warranted. Saccharification and fermentation of pretreated softwood was investigated in demonstration-scale experiments with 10 m^3^ bioreactors using an LPMO-containing cellulase preparation, a xylose-utilizing yeast, and either simultaneous saccharification and fermentation (SSF) or hybrid hydrolysis and fermentation (HHF) with a 24-hour or 48-hour initial phase and with 0.15 vvm aeration before addition of the yeast. The conditions used for HHF, especially with 48 h initial phase, resulted in better glucan conversion, but in poorer ethanol productivity and in poorer initial ethanol yield on consumed sugars than the SSF. In the SSF, hexose sugars such as glucose and mannose were consumed faster than xylose, but, in the end of the fermentation >90% of the xylose had been consumed. Chemical analysis of inhibitory pretreatment by-products indicated that the concentrations of heteroaromatic aldehydes (such as furfural), aromatic aldehydes, and an aromatic ketone decreased as a consequence of the aeration. This was attributed mainly to evaporation caused by the gas flow. The results indicate that further research is needed to fully exploit the advantages of LPMO without compromising fermentation conditions.

## 1 Introduction

The potential of production of ethanol and other commodities from cellulosic feedstocks has received great attention as an alternative to the production of fuels and chemicals from fossil resources (Kaparaju et al., 2009). There are several approaches to biorefining of lignocellulosic biomass. One approach is hydrothermal pretreatment followed by enzymatic saccharification of cellulose, fermentation of sugars, and valorization of the lignin-rich residue, i.e., the hydrolysis lignin (Martín et al., 2022). In such a process, the enzymatic saccharification is a key step for achieving high product yields. Enzyme consumption demands careful attention because of the costs associated with using enzyme preparations, which is a limiting factor for commercialization of the technology (Rosales-Calderon et al., 2019).

Conventional enzymatic saccharification of cellulose is based on hydrolytic enzymes, particularly endoglucanases, cellobiohydrolases, and β-glucosidases (Van Dyk and Pletschke, 2012). One of the barriers for conventional cellulose-degrading enzymes is their limited access to much of the cellulose due to the highly ordered cellulose fibrils (Arantes and Saddler, 2011). The degradation of cellulose can, however, be boosted through the action of enzymes providing auxiliary activities (AA), such as lytic polysaccharide monooxygenase (LPMO) of the AA9 family [formerly glycoside hydrolase family 61 (GH61)] (Hemsworth et al., 2015; Chylenski et al., 2019; Ipsen et al., 2021; Gandla et al., 2022). LPMO, which is a mononuclear copper enzyme, has become a common component of commercial enzyme cocktails because of its ability to synergistically work together with conventional cellulases in the saccharification of cellulose. By oxidative cleavage of glycosidic bonds, LPMO creates more substrate for other enzymes, thus contributing to higher sugar yields (Hemsworth et al., 2015; Chylenski et al., 2019; Ipsen et al., 2021; Gandla et al., 2022). The oxidation of cellulose by LPMO typically results in chain cleavage either at the C1 or the C4 positions of the sugar ring (Cannella et al., 2012; Chylenski et al., 2017; Chylenski et al., 2019; Gandla et al., 2022). LPMO-mediated C1 oxidation leads to the formation of a lactone, whereas C4 oxidation leads to formation of a ketoaldose (Chylenski et al., 2019; Gandla et al., 2022).

LPMO requires an oxidant, such as molecular oxygen, and a reductant for its catalytic activity. Various electron donors may serve as reductants (Hemsworth et al., 2015; Chylenski et al., 2019; Gandla et al., 2022), ranging from small molecules, such as ascorbic acid (Vaaje-Kolstad et al., 2010), to very large molecules, such as fungal cellobiose dehydrogenase (CDH) (Phillips et al., 2011). The various proposed electron donors also include lignin. Both lignin in the pretreated solids and lignin-degradation products in the liquid fraction have been found to serve as efficient reductants in LPMO-supported enzymatic saccharification of cellulose (Tang et al., 2022).

Saccharification and fermentation can be accomplished using different process configurations (Gandla et al., 2022). These include separate hydrolysis and fermentation (SHF) and simultaneous saccharification and fermentation (SSF). In SHF, saccharification and fermentation are performed sequentially in a two-step process, whereas in SSF both steps are combined into one (Takagi et al., 1977; Wyman et al., 1992; Olofsson et al., 2008). In SSF, the temperature is mandated by the microorganism and is typically not more than 35°C, which implies that the enzymatic saccharification is performed at suboptimal conditions (Olofsson et al., 2008). However, even if optimal temperature for enzymatic saccharification was used (typically 45°C–55°C), as in SHF, the reaction would be affected by end-product inhibition in which the sugars formed from hydrolysis of polysaccharides inhibit the cellulolytic enzymes (Xiao et al., 2004). End-product inhibition would not be a problem in SSF due to the consumption of sugar by the fermenting microorganism (Olofsson et al., 2008). Previous studies in which older generations of cellulase preparations were used typically resulted in higher yields of ethanol when using the SSF configuration (Öhgren et al., 2007; Tomás-Pejó et al., 2008, Erdi et al., 2010). However, the preferred processing strategy could be affected by the use of LPMO-containing enzyme preparations (Cannella and Jørgensen, 2014). Hybrid hydrolysis and fermentation (HHF) is a potential alternative, in which there is an initial enzymatic saccharification step that is followed by a lowering of the temperature (typically 30°C–35°C) and an addition of yeast.

Yeast fermentation is a metabolic process wherein yeast cells convert sugars into alcohol and carbon dioxide in the absence of oxygen. *Saccharomyces cerevisiae*, commonly known as baker’s yeast, is widely utilized in industrial bioethanol production. One notable characteristic of *Saccharomyces cerevisiae* is its efficient conversion of sugars to ethanol under both anaerobic and aerobic conditions. Even in the presence of oxygen, a situation where respiration could occur, *S. cerevisiae* exhibits a preference for alcoholic fermentation. This tendency is particularly evident at high glucose concentrations, a phenomenon known as the Crabtree effect (De Deken, 1966). Yeasts displaying this trait are referred to as Crabtree-positive yeasts. This metabolic feature plays a significant role in various industrial applications, especially in processes where ethanol production is desired.

In previous work on LPMO-supported saccharification of cellulose, we investigated the role of lignin and lignin-degradation products as reductants, the benefits of aeration for saccharification when LPMO is present, and how varying solids loadings and enzyme dosages affected reactions (Tang et al., 2022; Tang et al., 2023). Even though the benefits of aeration for saccharification were shown clearly in these studies, the question remains whether those benefits would outweigh benefits offered by the SSF approach, such as reduced end-product inhibition. To address that issue, we compared two different process configurations: a conventional SSF approach, without any aeration to support the LPMO reaction, and an HHF approach. The HHF approach consisted of a primary phase, with aeration and a higher temperature to suit the enzymatic saccharification reaction, followed by a secondary phase, without aeration and a lower temperature to suit the microbial fermentation process. Experiments were performed in a demonstration-scale facility with 10 m^3^ bioreactors using softwood pretreated through continuous steam explosion with sulfur dioxide as catalyst. The experiments differ from previous studies in the area in several ways: (*i*) the conditions used during the reactions, such as inclusion of aeration and the use of state-of-the-art enzyme preparation and xylose-fermenting *S. cerevisiae* yeast, (*ii*) the use of a demonstration-scale facility providing industrial-like conditions, (*iii*) the use of a softwood substrate pretreated through continuous steam explosion with sulfur dioxide as catalyst, (*iv*) the direct comparison of the SSF and the HHF approaches, and (*v*) the extensive analysis of potentially inhibiting by-products from the pretreatment and their fate during the initial phase of an HHF process with aeration. Besides the objective to address the trade-off between boosting LPMO catalysis through aeration in an HHF approach and minimizing end-product inhibition by using an SSF approach, the investigation also addresses potential effects of aeration on specific fermentation inhibitors and inhibition of *S. cerevisiae* yeast. Investigations in this area can offer guidance on the design of biochemical conversion processes by shedding light on benefits and drawbacks associated with different process configurations and reaction conditions.

## 2 Materials and methods

### 2.1 Pretreatment

Lignocellulosic slurry was produced in the Biorefinery Demo Plant (BDP) by Sekab E-Technology AB (Örnsköldsvik, Sweden) using continuous steam explosion with sulfur dioxide as catalyst. The biomass was sawdust of debarked Norway spruce (*Picea abies*). The approx. residence time was 12 min, the temperature was 195°C, the loading of sulfur dioxide was 0.3 kg/h, and the TS (total solids) of the resulting slurry was 25% (w/w).

### 2.2 Bioreactor experiments

Demonstration-scale experiments were carried out in 10 m^3^ stirred-tank bioreactors in the BDP using a working volume of 4 m^3^. Three experiments were performed: SSF, HHF1, and HHF2. In SSF, both enzymes and yeast were added in the beginning and there was no aeration, and the temperature was kept at 30°C throughout the reaction. In HHF1 and HHF2, there was an initial stage at 52°C with enzyme and aeration (0.15 vvm). After 24 h (HHF1) and 48 h (HHF2), aeration was discontinued, yeast was added, and the temperature was lowered to 30°C. The aeration rate (0.15 vvm) was chosen on basis of previous experiments (Tang et al., 2023) and practical considerations with respect to the available equipment. The question of the duration of the initial phase of HHF was addressed by including two different time periods (24 and 48 h) in the experimental set-up and comparing them with each other.

In all three experiments, the slurry was diluted to a final concentration of 12.5% (w/w) SS (suspended solids). The pH of the slurry was adjusted to 5.2 with an 18% aqueous solution of NaOH. For detoxification, a freshly prepared aqueous solution of sodium sulfite was added to a concentration of 10 mM and incubated for 10 min. The enzyme dosage was 4% w/w (i.e., 0.04 kg Cellic CTec3 from Novozymes, Bagsværd, Denmark) per kg SS. Before adding the yeast (*S. cerevisiae* CelluX™4, Leaf, Marcq-en-Barœul Cedex, France) to the bioreactors, it was reconditioned by incubation in 10 L sterile water for 30 min at 30°C. The yeast inoculum dosage was 1 kg/m^3^ final reaction mixture and 350 mL VitaHop was added as protection against bacterial growth during the fermentation process.

### 2.3 Analysis of solid fractions

#### 2.3.1 Compositional analysis of solid fractions

Two-step acid hydrolysis with sulfuric acid (TSSA) was used for determination of carbohydrates and lignin in the solid fractions of the reaction mixtures. After washing of the solid phase with deionized water, compositional analysis was conducted based on the protocol NREL/TP-510-42618 (Sluiter et al., 2012) with some modifications. Instead of HPLC (high-performance liquid chromatography), HPAEC-PAD (high-performance anion-exchange chromatography with pulsed amperometric detection) was used for the analysis of monosaccharides (further described in Section 2.4.1). Prior to the HPAEC-PAD analysis, the samples were diluted with ultra-pure water and filtered through 0.20 µm nylon membranes. Acid-soluble lignin (ASL) was determined at λ 240 nm using a UV-1800 spectrophotometer (Shimadzu, Kyoto, Japan). Acid-insoluble lignin (Klason lignin) was determined gravimetrically by using glass crucibles with integral glass sintered discs (Pyrex 2, porosity 10–16 µm). All analyses were conducted in triplicates.

#### 2.3.2 Pyrolysis-gas chromatography/mass spectrometry (Py-GC/MS)

The lignin-carbohydrate fraction of pretreated solids was analyzed using Py-GC/MS. The analysis was performed at the Biopolymer Analytical Platform (BAP) of the KBC Chemical-Biological Center (Umeå, Sweden). The method has been described in detail by Gerber et al. (2016).

#### 2.3.3 Calculation of glucan conversion

The fraction of glucan converted during the enzymatic saccharification and microbial fermentation (ΔGlc) was calculated based on the assumption that the lignin content was not changed. The equation was:

$$∆Glc\;\left(\%\right)=\left(1‐\left[\left(\frac{{\mathrm{Glc}}_{t1}\times L{\text{ig}}_{t0}}{{\mathrm{Lig}}_{t1}\;}\;\right)/G{\text{lc}}_{t0}\right]\right)\times 100$$ (1)

In Eq. 1, Glc_t0_ refers to the fraction of glucan in the beginning of the enzymatic saccharification reaction (t0), Glc_t1_ refers to the fraction of glucan at the sampling time point t1, Lig_t0_ refers to the fraction of lignin in the beginning of the enzymatic saccharification reaction, and Lig_t1_ refers to the fraction of lignin at time point t1.

### 2.4 Analysis of liquid fractions

#### 2.4.1 Analysis of monosaccharides using HPAEC-PAD

The HPAEC-PAD separation system used for quantification of monosaccharides (Dionex ICS-6000) was equipped with a CarboPac PA1 (4 mm × 250 mm) separation column with a (4 mm × 50 mm) guard column and an electrochemical detector (Thermo Scientific, Waltham, MA, United States). The column oven was set at 30°C. The separation of samples was conducted using ultra-pure water (Eluent A) for 25 min at a flow rate of 1 mL/min. Before that, the column was equilibrated by using a mixture consisting of 60% Eluent B (an aqueous solution of 300 mM sodium hydroxide) and 40% Eluent C (an aqueous solution of 200 mM sodium hydroxide and 170 mM sodium acetate). All samples were diluted with ultra-pure water and were filtered through 0.20 µm nylon membrane filters (Merck Millipore Ltd., Cork, Ireland). External calibration standards in the range of 0.5–30 mg/L were used for the quantification of monosaccharides. Each sample was analyzed in triplicates. Data evaluation was performed using the Chromeleon 7.1 software (Thermo Scientific).

#### 2.4.2 Analysis of furan aldehydes using HPLC

Quantification of the furan aldehydes furfural and HMF (5-hydroxymethylfurfural) was carried out using a Thermo Scientific Ultimate 3000 HPLC system (Dionex Softron GmbH, Germany) equipped with a UV detector. The separation was conducted on a Zorbax RRHT SB-C18 column (3.0 × 50 mm, 1.8 µm particle size) at a flow rate of 0.6 mL/min. Gradient elution was performed by using a mixture of Eluent D [an aqueous solution of 0.1% (v/v) formic acid] and Eluent E [0.1% (v/v) formic acid in acetonitrile]. The separation was conducted with 3% Eluent E for 3 min followed by a 4 min cleaning step with 20% of Eluent E, and finally the column was equilibrated with 3% Eluent E for another 4 min. The absorption at 282 nm was monitored and the temperature of the column oven was 40°C. An external calibration standard in the range 5 μM–250 µM was used for quantification and the Chromeleon 7.1 software was used for data evaluation.

#### 2.4.3 Analysis of ethanol and aliphatic acids using HPLC

Ethanol and aliphatic acids (formic acid, acetic acid, and levulinic acid) were analyzed using a Thermo Scientific Ultimate 3000 HPLC system (Dionex Softron GmbH, Germany) equipped with a refractive index detector (RID). The separation was performed on an Aminex HPX-87H column (Bio-Rad Laboratories AB, Solna, Sweden) with an eluent consisting of an aqueous solution of 0.005 M H_2_SO_4_ at a flow rate of 0.6 mL/min. The temperature of the column oven and detector was 55°C. The external calibration curve used for quantitation of the aliphatic acids was in the range 2 mM–250 mM, whereas the external calibration curve for quantitation of ethanol was in the range of 0.5 g/L to 25 g/L.

The calculations related to ethanol fermentation (volumetric ethanol productivity, Q (Eq. 2) and ethanol yield on consumed sugar (Eq. 3), Ycon) were:

$${Q\;(g\;L}^{‐1}{\;h}^{‐1})=\frac{\;C_{\text{EtOH}}}{t1\;‐\;\text{tf}0}$$ (2)

$$Y_{\text{con}}\;\left({g\;g}^{‐1}\right)=\frac{C_{\text{EtOH}}}{C_{\text{pre}‐\text{sug}}+{\;C}_{\text{es}‐\text{sug}}‐\;C_{\text{sug}}}$$ (3)

The volumetric ethanol productivity (Q) refers to grams of ethanol per liter and hour fermentation time (i.e., the time period since the yeast inoculum), C_EtOH_ is the ethanol concentration in grams per liter at sampling time point t1 hours, and tf0 is the time point in hours of the start of the fermentation. The yield on consumed sugar (Y_con_) refers to grams of ethanol per grams of consumed main fermentable sugars (glucose, mannose, and xylose), C_EtOH_ is the ethanol concentration in g L^−1^ at sampling time point t1, C_pre-sug_ is the concentration in g L^−1^ of the main fermentable sugars derived from the pretreatment in the beginning of the enzymatic saccharification (t0), C_es-sug_ is the concentration in g L^−1^ of the main fermentable sugars produced from the solid phase during the enzymatic saccharification reaction (between t0 and t1, and calculated based on the composition of the solid fraction assuming that the amount of lignin in the reaction mixture was constant), and C_sug_ is the concentration in g L^−1^ of the main fermentable sugars in the reaction mixture at sampling time point t1.

#### 2.4.4 Determination of pretreatment by-products using LC-MS/MS

Determination of formaldehyde, acetaldehyde, 4-hydroxybenzaldehyde, coniferyl aldehyde, vanillin, benzoquinone, *p*-coumaraldehyde, and acetovanillone was conducted using ultra-high performance liquid chromatography-electrospray ionization-triple quadrupole-mass spectrometry (UHPLC-ESI-QqQ-MS). 2,4-Dinitrophenylhydrazine (DNPH) was used for derivatization of analytes using a previously described approach (Ilanidis et al., 2021). The instrument used was an Agilent 1290 Infinity system equipped with an Agilent 6490 TripleQuad mass spectrometer with electrospray ionization (ESI) in negative mode. The following parameters were used: gas temperature, 290°C; gas flow, 20 L/min; sheath gas temperature, 400°C; sheath gas flow, 12 L/min. The separation was performed on a 2.1 mm × 50 mm Kinetex 1.7 µm biphenyl 100 Å column (Phenomenex, Torrance, California, United States) operating at 30°C at a flow rate of 0.3 mL/min. The eluent was a mixture of aqueous 0.1% (v/v) formic acid (Eluent F) and three-to-one (by volume) solution of acetonitrile and 2-propanol mixed with 0.1% formic acid (Eluent G). The MassHunter quant software was used for data evaluation (Agilent Technologies).

Determination of total phenolics (TPC, total phenolic content) was conducted using Folin-Ciocalteu’s reagent (Singleton et al., 1999). Vanillin was used as the calibration standard. Mixtures were incubated for 40 min at 20°C. The absorbance at λ 760 nm was measured by using a BioTek Epoch Microplate Spectrophotometer (Agilent, Santa Clara, CA, United States). Triplicate analyses were conducted.

Total aromatic content (TAC) was measured at λ 280 nm using a UV-1800 spectrophotometer (Shimadzu, Kyoto, Japan). The dilution factor was 500. TAC covers both aromatics (phenolic and non-phenolic aromatics) and heteroaromatics (such as HMF and furfural) (Wang et al., 2018). The analysis was conducted in triplicate.

Total carboxylic acid content (TCAC) was determined by titration from pH 2.8 to pH 7.0 using a 200 mM aqueous solution of sodium hydroxide (Wang et al., 2018).

#### 2.4.5 Analysis of LPMO oxidation products by HPAEC

Gluconic acid (C1 oxidation product) and a tentative C4 oxidation product from LPMO-catalyzed reactions were analyzed using an ICS-5000 HPAEC system with PAD (Dionex). The separation system consisted of a CarboPac PA1 column connected with an electrochemical detector using a flow rate of 1 mL/min for 20 min. The gradient elution included: 1.5 min with an aqueous solution of 0.3 M sodium hydroxide (Eluent B), 15.5 min with an aqueous solution of a mixture of 0.3 M sodium hydroxide and 0.5 M sodium acetate (Eluent H), ultra-pure water was the Eluent A. The column was regenerated by using Eluent G for 3 min before the next sample injection. The data evaluation was performed using Dionex Chromeleon 7.1.

## 3 Results and discussion

### 3.1 Compositional analysis of the pretreated biomass

Compositional analysis using TSSA revealed that the pretreated solids (i.e., values at 0 h) exhibited 53%–54% glucan and 44%–46% total lignin (including Klason lignin and acid-soluble lignin ASL) (Table 1). Remarkably, no carbohydrates other than glucan were detected. This observation strongly indicates that the pretreatment conditions were severe, resulting in complete removal of hemicellulosic carbohydrates. Untreated spruce wood consists of 27.4% lignin, 41.7% cellulose, and 28.3% carbohydrates other than cellulose (Sjöström, 1993). Thus, untreated spruce wood would be expected to have a carbohydrate:lignin ratio of 2.6, whereas the pretreated spruce exhibited a carbohydrate:lignin ratio of 1.1–1.2 (Table 1). The lower carbohydrate:lignin ratio as well as the increase of the fractions of both glucan and lignin compared to untreated spruce wood can be explained by the removal of the hemicelluloses.

**TABLE 1:** Chemical composition of solid fractions in reaction mixtures.

Analysis and constituent		SSF 0 h	SSF 24 h	SSF 48 h	SSF 96 h	HHF1 0 h	HHF1 24 h	HHF1 48 h	HHF1 96 h	HHF2 0 h	HHF2 24 h	HH2 48 h	HHF2 96 h
TSSA^a^	Glucan	53.0 (0.8)	46.8 (1.8)	46.9 (1.6)	40.2 (4.2)	54.4 (0.3)	40.9 (0.4)	39.2 (0.5)	37.0 (1.8)	53.3 (1.2)	38.1 (1.6)	37.1 (0.8)	33.9 (4.7)
	Xylan	ND	ND	ND	ND	ND	ND	ND	ND	ND	ND	ND	ND
	Mannan	ND	ND	ND	ND	ND	ND	ND	ND	ND	ND	ND	ND
	Arabinan	ND	ND	ND	ND	ND	ND	ND	ND	ND	ND	ND	ND
	Galactan	ND	ND	ND	ND	ND	ND	ND	ND	ND	ND	ND	ND
	Klason lignin	42.6 (1.1)	44.3 (1.1)	46.9 (0.8)	49.8 (0.9)	40.3 (1.0)	52.8 (0.5)	50.9 (2.5)	53.3 (1.1)	39.9 (0.9)	48.6 (2.8)	52.5 (2.0)	51.9 (3.3)
	ASL^c^	4.0 (1.0)	3.9 (0.1)	3.7 (0.1)	3.8 (0.1)	3.9 (0.2)	3.6 (0.1)	3.7 (0.2)	3.6 (0.2)	4.0 (0.1)	3.7 (0.1)	4.2 (0.3)	3.9 (0.1)
	Total lignin^d^	46.5 (1.0)	48.2 (1.1)	50.6 (0.8)	53.6 (0.9)	44.2 (1.0)	56.1 (0.1)	54.6 (2.5)	56.9 (1.0)	44.0 (0.8)	50.7 (0.3)	56.7 (2.3)	55.8 (3.3)
	Carb./lignin^e^	1.1 (0.1)	1.0 (0.1)	0.9 (0.1)	0.7 (0.1)	1.2 (0.1)	0.7 (0.1)	0.7 (0.1)	0.6 (0.1)	1.2 (0.1)	0.7 (0.1)	0.7 (0.1)	0.6 (0.1)
Py-GC/MS^b^	Carbohydrates	69.8 (0.4)	68.1 (1.9)	69.9 (1.0)	65.4 (1.0)	68.4 (0.1)	59.1 (1.1)	60.0 (0.2)	60.4 (0.6)	70.1 (1.8)	60.5 (1.0)	56.9 (0.6)	56.9 (0.6)
	G lignin	19.9 (0.3)	21.1 (0.1)	19.6 (0.7)	23.7 (0.7)	20.8 (0.3)	29.3 (1.3)	27.6 (0.2)	27.5 (0.2)	20.2 (1.4)	27.8 (0.4)	32.1 (0.8)	30.9 (0.6)
	H lignin	0.9 (0.1)	1.1 (0.1)	1.0 (0.1)	1.2 (0.1)	0.9 (0.1)	1.3 (0.1)	1.4 (0.1)	1.4 (0.1)	1.0 (0.1)	1.3 (0.1)	1.5 (0.1)	1.5 (0.1)
	Total lignin^d^	22.7 (0.3)	24.3 (0.2)	22.5 (0.8)	27.1 (0.7)	23.8 (0.3)	33.4 (1.2)	31.8 (0.1)	31.6 (0.4)	23.1 (1.5)	32.0 (0.9)	36.6 (0.7)	35.5 (0.5)
	Carb./lignin^e^	3.2 (0.1)	2.9 (0.1)	3.1 (0.2)	2.5 (0.1)	2.9 (0.1)	1.8 (0.1)	1.9 (0.1)	2.0 (0.1)	3.1 (0.3)	1.9 (0.1)	1.6 (0.1)	1.6 (0.1)

^a^ Two-step treatment with sulfuric acid. Values are averages of six measurements (triplicate reactions and duplicate analyses of each sample). Values are given as mass fractions in percent dry weight with standard deviations in parentheses.

^b^ Values are peak area fractions in percent according to the method described by Gerber et al. (2016). G, guaiacyl; H, *p*-hydroxyphenyl. Standard deviations in parentheses.

^c^ ASL, acid-soluble lignin.

^d^ Total lignin: For TSSA, Klason lignin plus ASL. For Py-GC/MS, G units, plus H units and other benzene derivatives (without OH group on aromatic ring), probably originated from lignin in plants.

^e^ The ratio of carbohydrate and total lignin. ND, not detected.

As expected for softwood (Sjöström, 1993; Ralph et al., 2004), the Py-GC/MS analysis showed that the lignin consisted predominantly of G (guaiacyl) units (Table 1). The Py-GC/MS analysis also suggested that the pretreated material predominantly (∼70%) consisted of carbohydrates and that the carbohydrate:lignin ratio was around three. Considering the carbohydrate:lignin ratio of untreated spruce and the removal of the hemicelluloses, the Py-GC/MS analysis overestimated the carbohydrate content of the pretreated material. Furthermore, the disparity compared to the TSSA values can also be explained by formation of pseudo-lignin. Pseudo-lignin is a Klason-lignin-positive aromatic substance that is not derived from native lignin. It is mainly formed from carbohydrates during thermal treatment under harsh conditions (Shinde et al., 2018; Martín et al., 2022; Yao et al., 2022). Pseudo-lignin is characterized as Klason lignin in the TSSA analysis (as it is acid resistant), but as carbohydrate in the Py-GC/MS analysis, a phenomenon that has been observed also in previous studies (Wang et al., 2018). Thus, data from the compositional analysis using TSSA and Py-GC/MS are consistent with quantitative removal of hemicelluloses and that the pretreatment conditions had been somewhat too severe, as conversion of carbohydrate to pseudo-lignin represents a yield loss with regard to sugars.

### 3.2 Analysis of sugars

Table 2 illustrates the evolution of monosaccharide concentrations during enzymatic saccharification and fermentation. In the beginning, mannose was the most abundant sugar (∼20 g/L), with glucose second (∼15 g/L) and xylose third (∼10 g/L). The concentrations of galactose (∼3 g/L) and arabinose (∼2 g/L) were low. This agrees with galactoglucomannans and arabinoglucuronoxylan being the most common hemicelluloses in softwood (Sjöström, 1993). As the *S. cerevisiae* yeast used in this study, CelluX™4, has been engineered for improved xylose utilization (Mokomele et al., 2023), it should be capable of fermenting all three main sugars in the hemicellulosic hydrolysate.

**TABLE 2:** Concentration (g L^−1^) of monosaccharides in the liquid fractions of the reaction mixtures.^a^

		0 h^b^	12 h^b^	24 h^b^	48 h^b^	72 h^b^	96 h^b^
SSF	Glucose	14.8 (0.3)	13.4 (0.2)	7.6 (0.1)	0.9 (0.1)	0.1 (0.1)	0.1 (0.1)
	Xylose	9.5 (0.2)	7.8 (0.1)	8.0 (0.1)	4.7 (0.1)	2.5 (0.1)	0.9 (0.1)
	Mannose	19.6 (0.4)	15.4 (0.2)	11.0 (0.4)	3.8 (0.1)	0.1 (0.1)	0.1 (0.1)
	Arabinose	2.1 (0.1)	2.2 (0.1)	3.0 (0.1)	1.9 (0.1)	1.4 (0.1)	1.2 (0.1)
	Galactose	3.4 (0.1)	2.8 (0.2)	3.2 (0.1)	1.6 (0.1)	ND	ND
HHF1	Glucose	14.6 (0.1)	31.3 (0.3)	50.4 (0.7)	33.6 (1.8)	20.2 (0.9)	4.9 (0.1)
	Xylose	9.7 (0.1)	8.8 (0.4)	8.7 (0.1)	7.2 (0.3)	7.2 (0.2)	5.7 (0.1)
	Mannose	20.7 (0.2)	17.7 (0.9)	16.9 (0.2)	13.2 (0.6)	12.8 (0.3)	8.7 (0.2)
	Arabinose	2.7 (0.1)	1.6 (0.1)	1.8 (0.1)	2.7 (0.1)	2.6 (0.1)	2.0 (0.1)
	Galactose	3.4 (0.1)	2.4 (0.1)	2.8 (0.1)	3.2 (0.2)	3.3 (0.1)	2.4 (0.1)
HHF2	Glucose	15.5 (0.3)	33.4 (1.8)	47.9 (0.4)	49.2 (0.2)	48.6 (1.3)	37.9 (0.6)
	Xylose	9.9 (0.2)	9.7 (0.2)	10.0 (0.1)	8.1 (0.1)	9.2 (0.1)	8.4 (0.1)
	Mannose	20.5 (0.4)	19.6 (0.3)	20.9 (0.1)	19.1 (0.6)	18.8 (0.2)	16.8 (0.1)
	Arabinose	2.2 (0.1)	1.6 (0.1)	1.8 (0.1)	1.6 (0.1)	1.9 (0.1)	1.6 (0.1)
	Galactose	3.6 (0.1)	2.6 (0.1)	2.7 (0.1)	2.5 (0.1)	3.5 (0.1)	3.1 (0.1)

^a^ Concentration of monosaccharides during saccharification and fermentation, where “0 h” is the start of the saccharification reaction.

^b^ The values shown are averages of technical triplicates. Samples withdrawn at 0 h were withdrawn before addition of enzyme, and samples at 24 h from HHF1 and at 48 h from HHF2 were withdrawn before addition of yeast. Standard deviations in parentheses. ND, not detected.

In the SSF reaction mixture, the three hexose sugars (glucose, mannose, and galactose) were reduced to very low levels after 48 h, and at 72 and 96 h the concentrations of these sugars were practically zero. For glucose, the reduction would include not only what was available in the hemicellulosic hydrolysate at the onset of the reaction, but also all glucose formed during the enzymatic saccharification of the cellulose. Xylose utilization was markedly slower than for the hexose sugars, but at the end of the fermentation (96 h) there was only a small fraction (9%) of the original concentration left. The arabinose concentration remained low (1–3 g/L) throughout the fermentation.

In HHF1, the glucose concentration rose rapidly up to 24 h, when the yeast was added (Table 2). After that, the glucose concentration decreased steadily, but, nevertheless, after 72 h of fermentation (i.e., at 96 h reaction time) around 5 g/L glucose still remained. There was a clear decrease in the concentrations of mannose and xylose, but in the end around 40% of the initial mannose and around 60% of the initial xylose still remained, which was a slower consumption than for the SSF in which <1% of the initial mannose and around 25% of the initial xylose remained after 72 h.

In HHF2, the glucose concentration rose rapidly up to around 50 g/L after 24 h, just as for HHF1, but then very little happened until 48 h, when the yeast was added (Table 2). This suggests a potential impact of end-product inhibition, and makes it difficult to justify extending the saccharification phase with 24 h compared to HHF1. After 48 h of fermentation (i.e., at 96 h reaction time), around 80% of the mannose and around 85% of the xylose still remained. This can be compared to the values after 48 h fermentation for SSF (∼20% mannose and ∼50% xylose) and HHF1 (∼60% mannose and ∼75% xylose), showing that HHF2 exhibited the slowest consumption of mannose and xylose.

### 3.3 Glucan conversion

**FIGURE 1:**
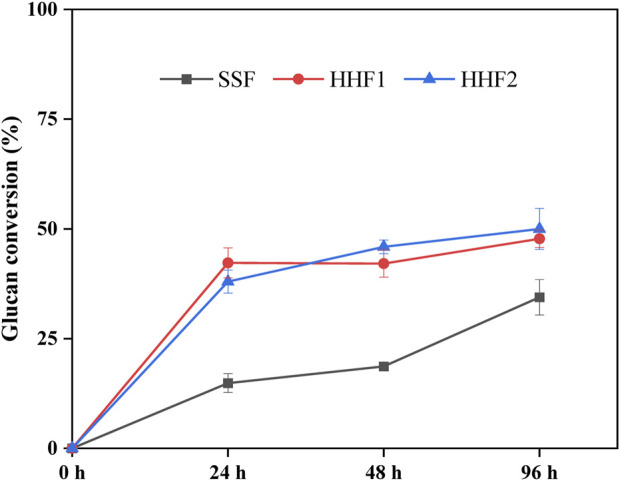
Glucan conversion based on analysis of the solid fractions in samples from SSF and HHF experiments. Calculation of glucan conversion was performed according to Eq. 1 in Subsection 2.3.3. The analysis was conducted in triplicate. Error bars show standard deviations.

Analysis of the glucose concentrations in the reaction mixtures is useful for following enzymatic saccharification in HHF1 up to 24 h and in HHF2 up to 48 h, but in order to assess enzymatic saccharification after addition of yeast calculations were made on basis of the composition of the solid phases after 24 h, 48 h, and 96 h. The glucan conversion in the reactions is shown in Figure 1. After 24 and 48 h, HHF1 and HHF2 had more than twice as high glucan conversion as the SSF. After 96 h, the glucan conversion in the SSF reaction had caught up to roughly 70% of the conversion in the HHF reactions.

The glucan conversion values were overall rather modest. This can be due to that the reactions were not completed after 96 h, and to the presence of pseudo-lignin (Hu et al., 2013) and other substances, such as phenols (Ximenes et al., 2011), that inhibit enzymatic saccharification reactions. Milder pretreatment conditions would have resulted in less pseudo-lignin and less phenols, and most probably better glucan conversion.

The superior glucan conversion in the HHF reactions compared to the SSF reaction, especially in the beginning, can tentatively be explained by aeration promoting the LPMO reaction and by the higher temperature in the HHF reactions. The results suggest that these factors were more important for achieving efficient saccharification than the reduction of sugars by the microorganism decreasing end-product inhibition. However, when the temperature in the HHF reactions is lowered to accommodate the yeast and the aeration is discontinued, these advantages would be lost and saccharification in the SSF reaction can at least partially catch up.

### 3.4 Oxidation products from LPMO reactions

**FIGURE 2:**
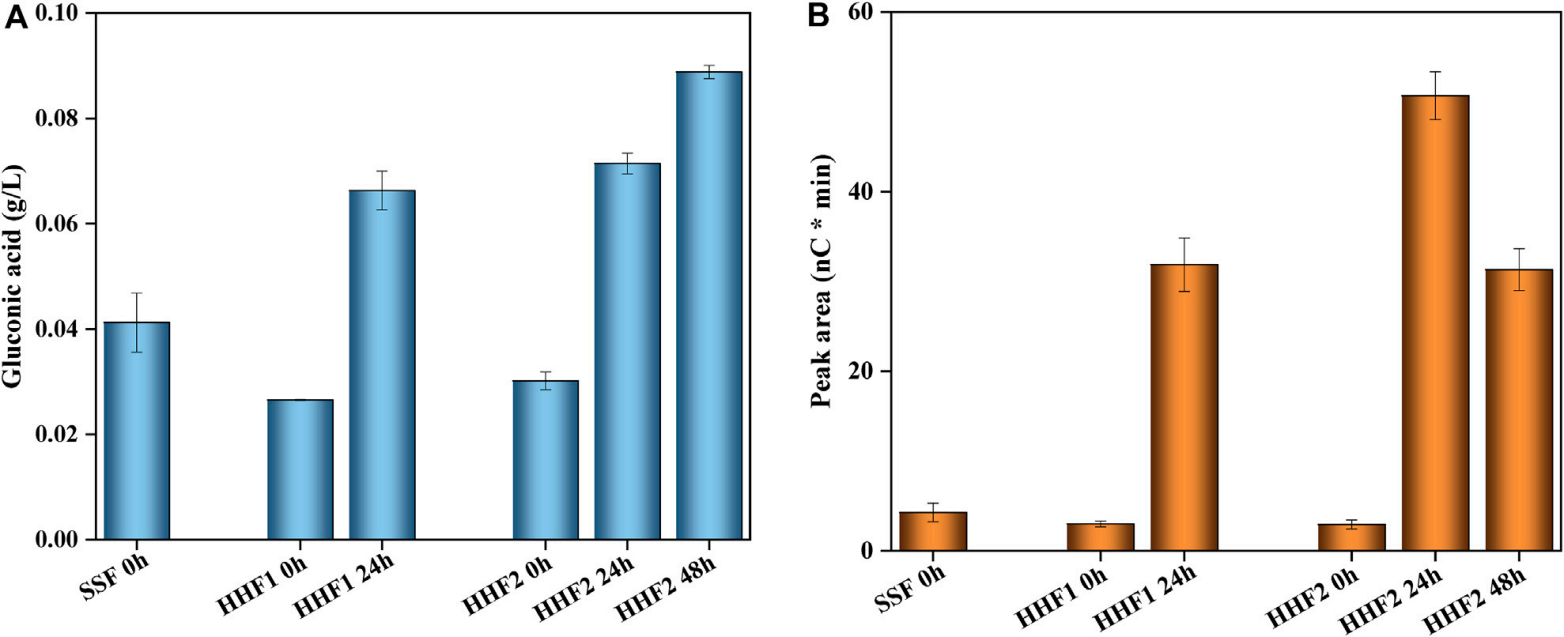
Changes in the concentrations of LPMO-related oxidation products in reaction mixtures from SSF and HHF experiments: **(A)** gluconic acid (g/L) and **(B)** tentative C4 product (arbitrary units). The analysis was conducted in triplicate. Error bars show standard deviations.

Besides common hydrolytic enzymes used for saccharification of cellulose, Cellic CTec3 contains LPMO, which contributes to the saccharification reaction provided that suitable oxidants (such as molecular oxygen in air) and reductants to support the LPMO-catalyzed reaction are available (Chylenski et al., 2017; Tang et al., 2022; Tang et al., 2023). Furthermore, formation of C1 and C4 oxidation products serves as an indication of LPMO activity (Cannella et al., 2012; Müller et al., 2015; Chylenski et al., 2019; Gandla et al., 2022; Tang et al., 2022). Figure 2 shows the analysis of oxidation products in reaction mixtures before the addition of yeast. Both HHF1 and HHF2 exhibited an increase in the concentration of gluconic acid, which is indicative of LPMO activity (Figure 2A). For HHF2, the increase of gluconic acid from 24 to 48 h was considerably smaller compared to the initial 24 h, which indicates that the LPMO reaction levelled off early on, which is consistent with the small changes observed for HHF2 in the monosaccharide analysis. With respect to the tentative C4 product, it was much higher in the HHF reactions after 24 and 48 h than in the beginning (Figure 2B). For HHF2, the concentration at 48 h was lower than that at 24 h. This is probably due to the inherent instability of the C4 product, making it prone to decomposition. The analysis of oxidation products supports that LPMO contributed to the saccharification reactions in the initial phase of the HHF experiments.

### 3.5 Analysis of pretreatment by-products

Both the microbial fermentation and the enzymatic saccharification can be affected by inhibiting by-products. Fermentation inhibitors include small aliphatic aldehydes, phenylic substances (i.e., phenolic and non-phenolic aromatics), benzoquinones, aliphatic acids, and furan aldehydes (Jönsson and Martín, 2016). Beyond end-product inhibition caused by monosaccharides (Xiao et al., 2004), enzyme inhibitors are less well characterized but include aromatic substances, such as phenolics (Ximenes et al., 2011; Jönsson and Martín, 2016; Zhai et al., 2016).

The concentrations of by-products in the reaction mixtures before addition of yeast are shown in Table 3. Regarding furan aldehydes, the initial concentrations were ∼11 mM (1.1 g/L) furfural and ∼10 mM (1.3 g/L) HMF. These concentrations are rather low and inhibitory effects, if any, would be small. The furan aldehydes decreased during aeration, especially furfural, which is more volatile than HMF (Larsson et al., 1999a; Tang et al., 2022).

**TABLE 3:** Pretreatment by-products before addition of yeast to the reaction mixtures.

	SSF (0 h)^a^	HHF1 (0 h)^a^	HHF1 (24 h)^a^	HHF2 (0 h)^a^	HHF2 (24 h)^a^	HHF2 (48 h)^a^
Furfural^b^	11.0 (0.1)	10.7 (0.2)	7.7 (0.1)	10.8 (0.1)	9.4 (0.1)	6.3 (0.1)
HMF^b^	10.7 (0.1)	10.3 (0.2)	8.4 (0.1)	10.5 (0.1)	9.9 (0.1)	8.2 (0.2)
Acetic acid^b^	77 (2)	74 (1)	71 (1)	74 (1)	75 (1)	70 (1)
Formic acid^b^	61 (2)	62 (3)	49 (1)	62 (3)	59 (2)	47 (1)
Levulinic acid^b^	4.4 (0.3)	4.0 (0.1)	3.8 (0.1)	4.2 (0.1)	4.2 (0.1)	4.1 (0.1)
Formaldehyde^c^	6.2 (0.6)	6.8 (0.3)	4.6 (0.1)	6.2 (0.7)	6.1 (0.1)	6.9 (0.2)
Acetaldehyde^c^	89 (2)	82 (2)	36 (1)	89 (6)	38 (2)	41 (1)
4-Hydroxybenzaldehyde^c^	19 (1)	12 (1)	4 (1)	11 (1)	9 (1)	4 (1)
Vanillin^c^	339 (2)	353 (7)	213 (5)	346 (4)	314 (3)	190 (7)
Coniferyl aldehyde^c^	92 (4)	101 (1)	50 (1)	95 (2)	81 (1)	50 (1)
Acetovanillone^c^	7.3 (0.4)	6.2 (0.1)	3.1 (0.2)	6.1 (0.2)	5.2 (0.1)	3.5 (0.2)
*p*-Coumaraldehyde^c^	1.4 (0.1)	1.3 (0.1)	0.8 (0.1)	1.3 (0.1)	1.1 (0.1)	0.8 (0.1)
Benzoquinone^c^	3.7 (0.1)	3.2 (0.1)	2.6 (0.1)	3.7 (0.1)	3.3 (0.1)	3.5 (0.1)
TPC^d^	3.5 (0.1)	3.5 (0.1)	3.4 (0.1)	3.5 (0.1)	3.7 (0.1)	3.2 (0.1)
TAC^e^	0.6 (0.1)	0.6 (0.1)	0.5 (0.1)	0.6 (0.1)	0.5 (0.1)	0.5 (0.1)
TCAC^f^	107 (4)	109 (2)	111 (2)	109 (1)	114 (5)	109 (2)

^a^ Mean values of technical triplicates. Standard deviations are shown in parentheses. Samples withdrawn at 0 h were withdrawn before addition of enzyme, and 24 and 48 h samples were withdrawn before addition of yeast.

^b^ Furans and aliphatic acids were analyzed using HPLC; concentrations in mM.

^c^ Determined using UHPLC-ESI-QqQ-MS; concentration of formaldehyde in mM and other concentrations in μM.

^d^ Total phenolics were analyzed using the Folin-Ciocalteu assay; concentrations in g/L.

^e^ Total Aromatic Content indicated as absorbance units at 280 nm (AU_280_) with a dilution factor of 500.

^f^ Total Carboxylic Acid Content (mM) determined using titration with sodium hydroxide.

The main aliphatic acids were acetic acid (∼75 mM) and formic acid (∼62 mM), whereas the levels of levulinic acid were low (∼4 mM). As the combined concentration would be higher than 100 mM (Table 3), there would probably be a slight inhibitory effect on yeast (Larsson et al., 1999b).

The concentration of formaldehyde was ∼6.2 mM (Table 3), which is expected to be strongly inhibitory to yeast (Cavka et al., 2015), whereas the concentration of acetaldehyde was ∼87 μM, which is well below inhibitory concentrations (Cavka et al., 2015). The concentration of acetaldehyde clearly decreased during aeration, whereas the changes in the concentration of formaldehyde were not consistent despite that these analyses were repeated.

Of the individual phenolic substances analyzed, both the phenolic aldehydes (4-hydroxybenzaldehyde, vanillin, coniferyl aldehyde, and *p*-coumaraldehyde) and the phenolic ketone (acetovanillone) decreased during aeration (Table 3). As the phenolic ketone decreased as much as many of the phenolic aldehydes, it is plausible that evaporation due to gas flow during aeration was the main mechanism, rather than chemical oxidation. Although benzoquinone has very high molar toxicity, the low concentrations observed (3–4 μM) probably did not affect the reactions.

The TPC was rather high (∼3.5 g/L) (Table 3) and might have affected both fermentation and enzymatic saccharification. Total Aromatic Compounds (TAC) includes aromatics, including phenolic compounds (both phenolic and non-phenolic aromatics) and heteroaromatics (e.g., furan aldehydes). The TAC and TCAC values were not very high and changes during aeration were small. There was perhaps a small decrease in TAC, probably caused by evaporation of furfural and phenols. Formation of LPMO oxidation products and oxidation of aldehydes to carboxylic acids might have contributed to a slight increase in TCAC.

In summary, many fermentation inhibitors decreased slightly during the aeration, probably mostly as a consequence of evaporation. The effects on formaldehyde need to be investigated further in the future.

### 3.6 Ethanol productivity and yield

The volumetric ethanol productivity and the ethanol yield based on consumed sugars were calculated and are presented in Table 4. The volumetric ethanol productivity reflects the rate of the ethanol production during the fermentation, and is dependent on factors such as the size of the inoculum, the access to nutrients, the redox conditions, and potential influence of fermentation inhibitors. The ethanol yield on consumed sugars shows the size of the fraction of fermentable sugars that was directed to ethanol production, with 0.51 g/g being the maximum value for conversion of glucose to ethanol. However, actual yields in fermentation processes are typically lower due to factors such as utilization of sugar for respiration and growth, and by-product formation.

**TABLE 4:** Ethanol concentration, ethanol productivity and ethanol yield during fermentation.^a^

	Ethanol concentration (g/L)				Volumetric ethanol productivity (Q) (g/L/h)				Ethanol yield on consumed sugars (Y)^b^			
	24 h	48 h	72 h	96 h	Q_24_	Q_48_	Q_72_	Q_96_	Y_24_	Y_48_	Y_72_	Y_96_
SSF	10.4 (0.1)	19.2 (0.7)	26.8 (0.1)	29.4 (0.1)	0.43 (0.01)	0.40 (0.01)	0.37 (0.01)	0.31 (0.01)	0.36 (0.02)	0.39 (0.01)	-	0.43 (0.01)
HHF1^c^	6.8 (0.1)	15.0 (0.7)	22.5 (0.2)	-	0.28 (0.01)	0.31 (0.01)	0.31 (0.01)	-	0.29 (0.07)		0.36 (0.01)	-
HHF2	2.8 (0.1)	4.8 (0.1)	9.3 (0.1)	11.1 (0.6)	0.12 (0.01)	0.10 (0.01)	0.13 (0.01)	0.12 (0.01)	-	0.23 (0.03)	-	

^a^ Mean values of technical triplicates. Standard deviations are shown in parentheses.

^b^ “Consumed sugars” refers to fermentable sugars, which include glucose, mannose, and xylose.

^c^ The total reaction time for HHF1 was 96 h, and as the fermentation started 24 h after enzymatic saccharification was initiated there is no data for 96 h fermentation time.

SSF consistently exhibited the highest volumetric ethanol productivity, with the highest value, 0.43 g/L/h, appearing already after 24 h (Table 4). That agrees well with data in Table 2, which shows that the glucose concentration was very low after 48 h and that the total sugar concentration was low (12.9 g/L) compared to the beginning of the fermentation (49.4 g/L). HHF1 exhibited the second highest values (∼0.3 g/L/h) and HHF2 the lowest values (∼0.1 g/L/h).

At a given fermentation time (24 or 48 h), the SSF reaction always showed higher ethanol yield on consumed sugars than the HHF reactions (Table 4). Also, for the reactions where data are available (SSF and HHF1), the yields increased with increasing fermentation time. The yield values ranged from 0.23 g g^−1^ for Y_48_ of HHF2 to 0.43 g g^−1^ for Y_96_ of SSF (Table 4), which is well below the theoretical maximum (0.51 g g^−1^).

Low ethanol yields on consumed sugar may be the result of utilization of sugars for other purposes than ethanolic fermentation, by-product formation, and, possibly, consumption of ethanol after depletion of sugar under aerobic conditions. Although *S. cerevisiae* is a Crabtree-positive yeast that produces ethanol even under aerobic conditions provided that sugar concentrations are sufficiently high, there is a risk that factors such as large bioreactor headspace and (for HHF reactions) aeration contributed to sugar being utilized for other purposes than ethanolic fermentation. The increasing ethanol yields at later time points (Table 4) are consistent with initial fermentation conditions not being optimal for ethanol production and that the fermentation conditions then improved over time.

### 3.7 Conclusion

Two process configurations, SSF and HHF with aeration during the first phase, were compared in an industrial-like setting using pretreated softwood devoid of hemicelluloses, an LPMO-containing enzyme preparation, and a xylose-fermenting *S. cerevisiae* yeast. Aeration to promote the LPMO reaction and using a relatively high temperature promoted glucan conversion in the HHF reactions, but the overall conversion was modest, probably mostly due to formation of pseudo-lignin. An extension of the yeast-less initial phase of the HHF reactions from 24 to 48 h had some beneficial effect on the saccharification as shown by higher glucan conversion values, but a negative impact on the ensuing fermentation process. Although HHF with aeration increased the glucan conversion, the positive effects were not sufficient to reach similar ethanol yield and productivity as was achieved with SSF. Detailed chemical analysis of the reaction mixtures suggested that the concentrations of most fermentation inhibitors decreased during aeration, probably mainly owing to evaporation effects resulting from the gas flow. However, further analyses of fermentation inhibitors, including formaldehyde, are warranted, especially as the HHF reactions exhibited relatively poor fermentability compared to the SSF reaction. Also, enzyme inhibitors, including potential inhibitors of LPMO, warrant further attention in the future. The observation of low values for ethanol yield on consumed sugar for the HHF reactions, especially during the first 48 h, suggest that redox environment and product formation during fermentation need to be addressed in future studies.

## Data Availability

The original contributions presented in the study are included in the article/supplementary material, further inquiries can be directed to the corresponding author.
